# Genome editing is revolutionizing biology

**DOI:** 10.1186/s13578-017-0162-6

**Published:** 2017-07-14

**Authors:** Yiping Qi

**Affiliations:** 1 Department of Plant Science and Landscape Architecture, University of Maryland, College Park, MD 20742 USA; 2 Institute for Bioscience and Biotechnology Research, University of Maryland, Rockville, MD 20850 USA

Throughout the very recent history of molecular biology, only a handful of ground breaking technologies have revolutionized biology across many fields. For example, polymerase chain reaction (PCR) invented in the 1980s offered a straightforward way to amplify DNA in vitro. This technology has been rapidly adopted to every molecular laboratory since. Obviously, it has numerous applications in basic science, agriculture and medicine. PCR technology was later expanded to detect and quantify RNA.

Next generation sequencing (NGS) technology is a second example of a revolutionizing technology. NGS includes multiple high-throughput sequencing platforms which are still undergoing rapid evolution. Consequently, DNA sequencing costs have been in steady decline, making NGS technology more and more accessible to ordinary researchers for a myriad of applications. NGS has been applied at all fronts of biology, from association mapping in basic science to medical diagnosis.

In recent years, genome editing has emerged as another revolutionizing technology that is currently transforming biology. Genome editing allows for rewriting endogenous DNA sequences, the blueprint of the cell. Achieving genome editing in eukaryotic cells often relies on sequence specific nucleases (SSNs). Easy-to-use SSNs were lacking throughout the early development of this technology. Development of facile transcriptional activator-like effector nucleases (TALEN) and clustered regularly interspaced short palindromic repeats (CRISPR) systems have made genome editing achievable in many laboratories. In this thematic issue on genome editing, two review articles are included. The first review by Chen et al. [1] summarizes the recent history of genome editing technologies in the worm model *Caenorhabditis elegans.* Pros and cons of different genome editing technologies are compared and discussed in this review. The second review by Malzahn et al. [2] describes recent advances in plant genome editing with TALEN and CRISPR systems. Genes that have been edited in plant species with both systems are summarized. These reviews give a substantial focus on CRISPR as it is the most versatile tool to be used for genome editing. These reviews highlight current obstacles in genome editing for worm and plant systems. Repurposing genome editing tools for transcriptional modulation are also discussed.

Like NGS, genome editing is experiencing constant evolution for improvement and diversified applications. Since genome editing requires the cooperation of SSNs and DNA repair pathways, the outcome of genome editing will likely be more dependent on cell types and organisms under study. A system that functions efficiently in worm cells may not be directly transferrable to plant cells, and vice versa. This should recruit a broad diversity of talent and skillsets into the field of developing and demonstrating innovative and effective genome editing tools and approaches across many biological systems. PCR amplifies DNA, NGS reads DNA and genome editing rewrites DNA. When these three revolutionizing technologies come together, the future is bright.
